# Effect of immunomodulatory agents on the response to COVID-19 vaccination among patients with neuromuscular diseases: A single center experience

**DOI:** 10.1097/MD.0000000000041606

**Published:** 2025-02-28

**Authors:** Jaylin Hsu, Perry B. Shieh

**Affiliations:** a Department of Neurology, University of California, Los Angeles, CA; b Department of Pediatrics, University of California, Los Angeles, CA.

**Keywords:** B-cell depleting therapies, Corticosteroids, COVID-19 antibody, IVIG, mycophenolate mofetil

## Abstract

Immunomodulatory agents, commonly used in autoimmune neuromuscular disorders, may significantly attenuate immunological response to vaccines. Yet, the degree to which different classes of these drugs suppress the immune system is unclear. This study aimed to characterize the response to the coronavirus disease 2019 (COVID-19) vaccines among our cohort of patients with neuromuscular diseases, including both patients who are and are not receiving immunomodulatory agents. This was a retrospective chart review of our single-center neuromuscular clinic patients who had undergone semi-quantitative COVID-19 antibody testing. A sum of 111 patients were initially identified, and 44 were excluded because of various reasons (e.g., COVID-19 infection, etc). The remaining 67 patients had undergone antibody testing after receiving one of the FDA-approved COVID-19 vaccines (2 doses of Moderna or Pfizer/BioNTech, or 1 of Janssen). A sum of 52 of these patients were receiving immunomodulatory treatments, and 15 were not. Patients were grouped based on their relative antibody response to vaccination, and the antibody responses of patients on each of the different immunomodulatory treatments were compared to those of patients not on any immunomodulation. Patients receiving B-cell depleting therapies demonstrated the weakest response to vaccination (*P* = .002), and those on mycophenolate mofetil also displayed a weaker response compared to patients not on immunomodulation (*P* = .045). Corticosteroids (*P* = .584) and intravenous immunoglobulin (*P* = .941) had minimal effect on COVID-19 antibody response. The degree to which a specific agent may affect a patient’s immune response to vaccines or infections may play a role in a clinician’s choice of treatment.

## 1. Introduction

Neuromuscular conditions, including autoimmune disorders in which the immune system is directed at the peripheral nervous system or muscles, result in debilitating muscle weakness and impairment. Conditions that fall under this category include myasthenia gravis, chronic inflammatory demyelinating polyneuropathy, and inflammatory myopathy. Immunomodulatory therapies are the mainstay of treatment in these patients. Alongside autoimmune disorders, some inherited neuromuscular disorders, such as Duchenne muscular dystrophy, may also be treated with immunomodulation, particularly with corticosteroids, which have anti-inflammatory effects that are known to be disease-modifying in these conditions.

Common immunomodulatory agents used to treat autoimmune neuromuscular disorders include rituximab and ocrelizumab, which are anti-CD-20 antibody agents^[1]^; mycophenolate mofetil, an anti-folate agent that suppresses T and B lymphocytes and their proliferation^[2]^; corticosteroids, such as prednisone and deflazacort, which lead to anti-inflammatory effects^[3]^; azathioprine, which inhibits purine synthesis as well as B and T cells^[4]^; and intravenous immunoglobulin (IVIG) that has a number of mechanisms, including but not limited the modulation of the humoral immune system and inhibition of monocyte effector mechanisms.^[5]^

While these agents have established clinical efficacy, previous studies have also shown that some of these agents may diminish the immunological response to vaccines.^[6,7]^ However, there is limited knowledge of the degree to which specific immunomodulatory agents affect the immune response, particularly in response to vaccination. The emergence of the coronavirus disease 2019 (COVID-19) pandemic and the fast development of vaccines have led to increased questions in this area. With a weakened immune system, patients on immunomodulation are positioned to have a weaker response to vaccinations, leading to significantly reduced protection against deadly diseases such as COVID-19. In medical settings, these patients often inquire about the degree to which these agents affect the immune response to vaccines as well as their ability to fight infections. The authors decided to provide insight into this question by reviewing patients’ antibody responses as a surrogate marker of immune response to the COVID-19 vaccination.

## 2. Materials and methods

### 2.1. Study design

We performed a retrospective chart review of patients in our neuromuscular clinic who had undergone semi-quantitative severe acute respiratory syndrome coronavirus 2 spike antibody immunoglobulin G tests (Quest Diagnostics test code 34499)^[8]^ at least 1 month after vaccination completion. Quest Diagnostics reported antibody levels as negative (<1.00 µg/mL), or positive (≥1.00 µg/mL), and titer values (>20.00 µg/mL) were beyond the level of quantification and thus reported as “>20.00 µg/mL.” IRB approval was obtained for this chart review (UCLA IRB #23-000186).

### 2.2. Patient selection

**Figure 1.**
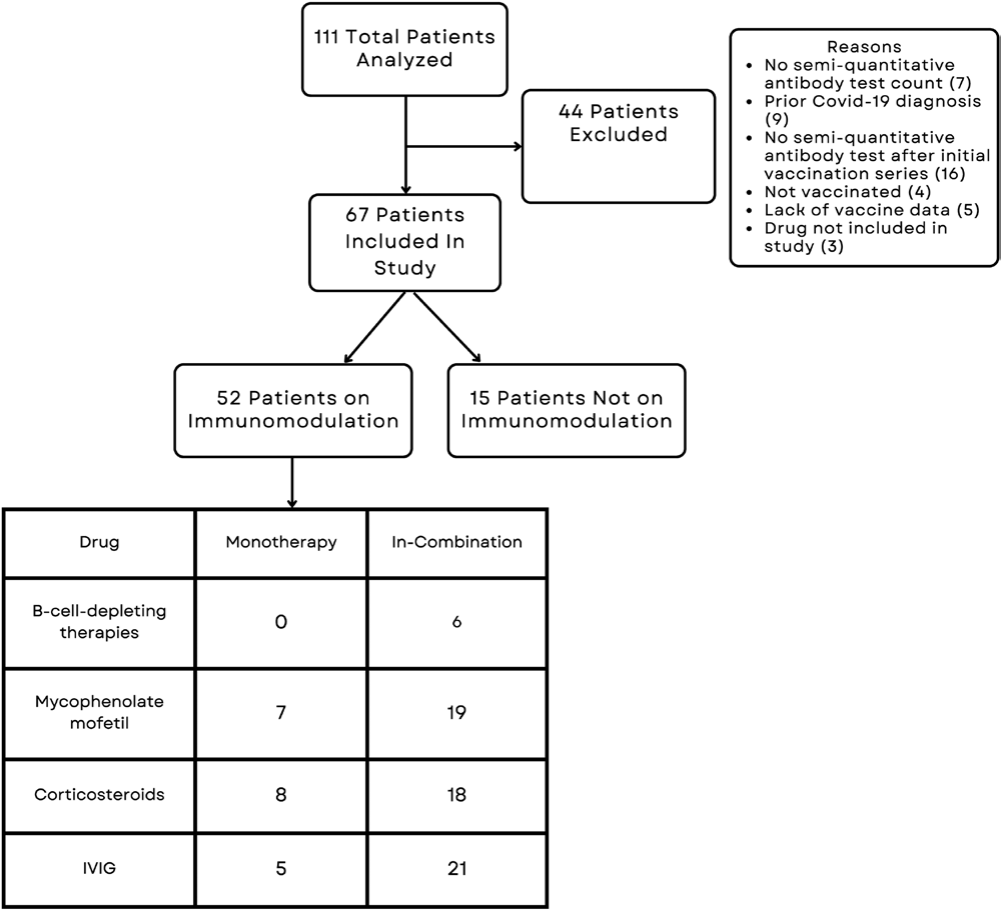
The selection process for this study. Monotherapy was defined as patients on only 1 immunomodulatory agent. In-combination was defined as patients on more than 1 immunomodulatory agent. IVIG = intravenous immunoglobulin.

A sum of 111 patients with autoimmune neuromuscular and inherited neuromuscular diseases (including myasthenia gravis, chronic inflammatory demyelinating polyneuropathy, immune-mediated necrotizing myopathy, dermatomyositis, Duchenne muscular dystrophy, limb–girdle muscular dystrophy, and spinal muscular atrophy) were initially selected for this retrospective analysis based on having had COVID-19 antibody testing. A sum of 44 patients were excluded from the primary analysis for the following reasons: their antibody testing did not provide a semi-quantitative value, they had a confirmed diagnosis of COVID-19 infection prior to antibody testing or vaccination, they had not received semi-quantitative antibody testing after the initial vaccination series, the patient had not undergone COVID-19 vaccination, their vaccination and/or antibody test dates were not available, or they were on an immunomodulatory agent that was not commonly used in our clinic cohort. Of the remaining 67 patients, 52 were on at least one of the commonly used immunomodulatory treatments in our clinic, and 15 were on no immunomodulatory treatments (see Table S1, Supplemental Digital Content, http://links.lww.com/MD/O405, which displays the diagnoses and treatments for these 67 patients). All patients in this cohort had completed the full COVID-19 vaccination series (Moderna or Pfizer/BioNTech or Janssen), and some had received booster doses. Among these 67 patients in the primary analysis cohort, the average (SD) time elapsed between vaccination and subsequent antibody testing was 124.32 (73.80) days for all patients included in this study. For each patient, the type of immunomodulatory agent(s) taken at the time of vaccination, type of vaccine, dates of vaccine series and booster shots, dates of antibody test(s), and results of the semi-quantitative antibody test(s) were collected. We included patients on B-cell depleting therapies (rituximab), which were given to patients with more severe myasthenia gravis, mycophenolate mofetil, corticosteroids (prednisone and deflazacort), and IVIG in this analysis. Figure 1 is a flow chart representing the initial overview of patients in the study.

### 2.3. Group description and comparison

All 67 patients were categorized as a responder, defined as a patient with a positive antibody level (≥1.00 µg/mL) after completing their vaccination series (2 injections for Moderna and Pfizer/BioNTech and 1 injection of Janssen), or a nonresponder, defined as a patient who had a negative antibody titer (<1.00 µg/mL) after the initial vaccination series.

The effect of individual immunomodulatory agents on antibody response was then analyzed by comparing the relative number of responders and nonresponders among patients taking a specific medication. We first examined patients on only 1 immunomodulatory treatment (i.e., monotherapy) and their relative responses. By excluding patients on combination therapy, the effect on antibody production could be confidently attributed to the single agent the patients were taking. Although this analysis was somewhat informative, the number of patients on monotherapy was small (n = 20). Thus, we expanded the patient pool to include patients on more than 1 immunomodulatory agent, allowing IVIG and corticosteroids as add-on therapy since these were found not to have a significant effect in the first analysis. Eculizumab was also allowed in the combination analysis based on prior studies that established that eculizumab has no significant effect on antibody production.^[9]^ The relative response of the patients on each of the different immunomodulatory agents was compared with the control population of patients not on immunomodulatory agents.

Subanalyses were then performed that grouped the responders and nonresponders into additional subcategories. The responders were separated into “strong responders” who demonstrated an antibody level > 20.00 µg/mL (out of the detectable limit range in semiquantitative antibody test result) and “weak responders” who exhibited an antibody level of 1.00 µg/mL to 20.00 µg/mL following the first vaccination series. The nonresponders were separated into “delayed responders” who presented no detectable antibodies succeeding the full vaccination series but ≥1.00 µg/mL after a booster dose and “poor responders” who had no detectable antibodies after at least 1 booster. Nonresponders who did not receive an additional vaccination dose or did not undergo subsequent semi-quantitative antibody retest were not included. To avoid selection bias, the subgroups were renormalized and represented as percentages. The same analyses were performed for patients not on any immunomodulation.

### 2.4. Statistical analyses

Statistical analysis was performed using the IBM SPSS Statistics Software V 30.0. To assess the relationship between the responders and nonresponders via the frequency of individuals on each of the 4 immunomodulatory agents compared to that of individuals not on immunomodulatory therapy, chi-square tests with asymptotic significance (2-sided) were run to calculate 4 distinct *P* values. Statistical significance was programmed to *P* < .05.

## 3. Results

Among the 15 patients in our cohort not on any immunomodulatory treatments, 87% (13/15) exhibited a response, while 13% (2/15) did not show a detectable antibody response. We considered these patients to be our “control” group against which we would compare the remaining patients who were on various immunomodulatory agents.

**Figure 2.**
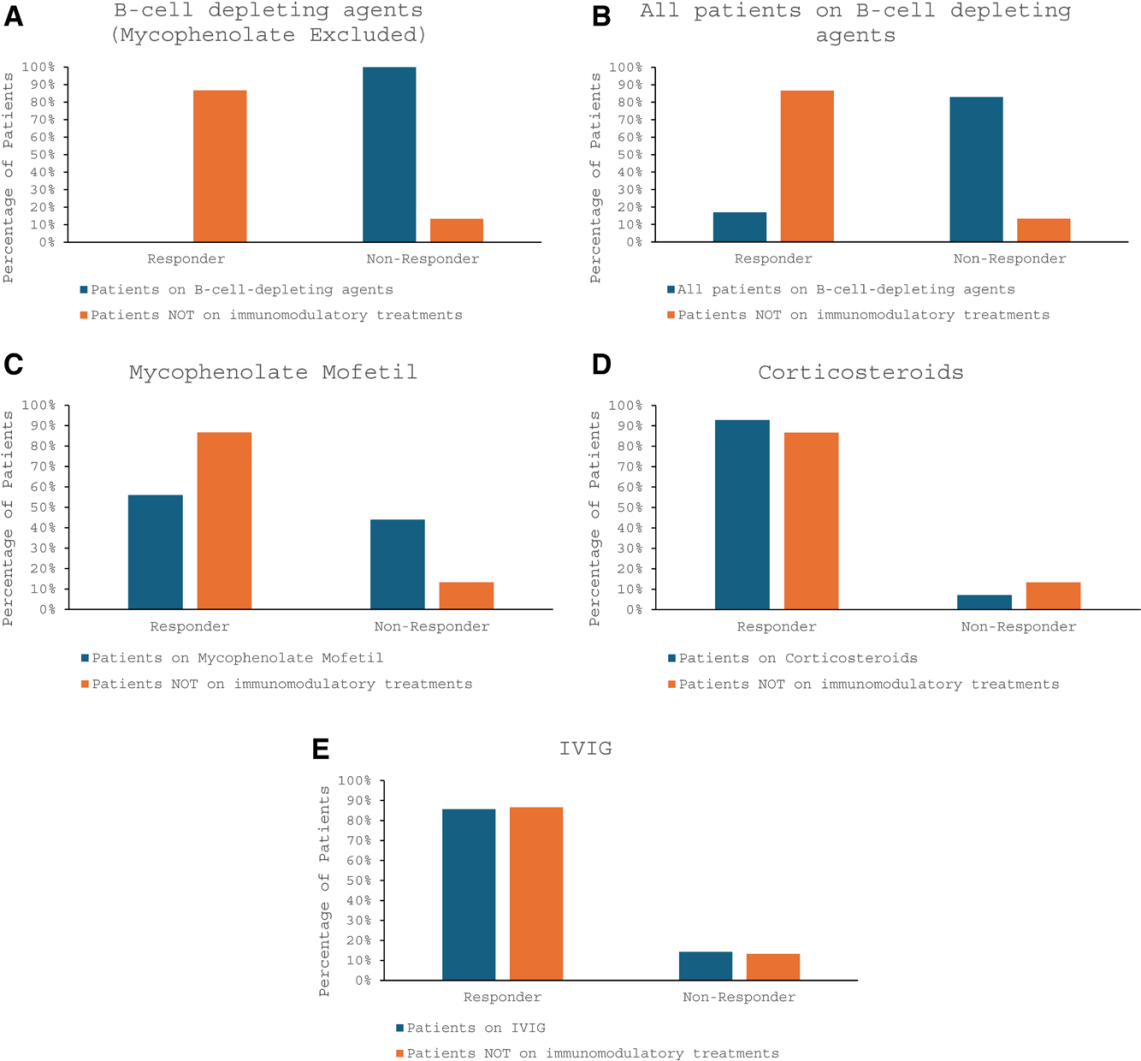
(A–E) Percent of patients who were responders and non-responders. Patients not on any immunomodulatory treatment (in orange) are compared with (A) patients on B-cell depleting agents (in blue, mycophenolate treated patients excluded), (B) patients on B-cell depleting therapies (in blue, no patients excluded), (C) patients on mycophenolate mofetil but not on B-cell depleting agents (in blue), (D) patients on corticosteroids and not on B-cell depleting agents and/or mycophenolate mofetil (in blue), (E) patients on IVIG and not on B-cell depleting agents and/or mycophenolate mofetil (in blue). IVIG = intravenous immunoglobulin.

There were no patients who had received B-cell depleting agents as monotherapy, but there were 4 patients who received B-cell depleting agents with only IVIG and/or corticosteroids. Among these 4 patients, all were classified as nonresponders. Because of the small number of patients in this analysis, we expanded the analysis to include all patients who received B-cell depleting agents. Among these patients, 17% (1/6) displayed a response, while 83% (5/6) were nonresponders. Not surprisingly, compared to patients not on immunomodulatory agents, B-cell depleting agents appear to dramatically attenuate antibody responses (*P* = .002; Fig. 2A, B).

Because of the significant effect of B-cell depleting therapies, patients who had received B-cell depleting therapies were excluded from the analysis of mycophenolate mofetil. Among these patients, 56% (14/25) were responders, while 44% (11/25) were nonresponders. Compared to patients not on immunomodulatory agents, mycophenolate mofetil appears to modestly increase delays in antibody responses (*P* = .045; Fig. 2C).

Because of the effect of mycophenolate on antibody response, we excluded these patients from the analysis of other agents. Among our patients taking corticosteroids but not on B-cell depleting therapies or mycophenolate, 93% (13/14) were responders, while 7% (1/14) were nonresponders. Compared to patients not on immunomodulatory agents, corticosteroids do not appear to significantly attenuate antibody responses to vaccines (*P* = .584; Fig. 2D).

Lastly, we carried out the same analysis of patients who were on maintenance IVIG infusions. Among the patients on IVIG but had not received B-cell depleting therapies or mycophenolate, 86% (12/14) were responders, while 14% (2/14) were nonresponders. Compared to patients not on immunomodulatory agents, IVIG does not appear to significantly diminish antibody responses to vaccines (*P* = .941; Fig. 2E).

Each analysis shown above was subjected to subgroup analyses (see Figures S2–S6, Supplemental Digital Content, http://links.lww.com/MD/O405, which display subgroup results) to provide additional detail to the immunological response profile.

Among the 67 patients included in the primary analysis, the average age of responders was 48.8 years, and of the nonresponders was 62.7 years.

## 4. Discussion

Neuromuscular patients on immunomodulation often ask about their risk of infection. As clinicians, we often discuss the risk-benefit analysis and the relatively low incidence of serious infections among our patients on these medications. Nonetheless, we also encourage the patient to exercise caution and limit exposure to infections.

This current study uses a semi-quantitative antibody assay as a surrogate measure of the immunological response to COVID-19 vaccination. The data provides some context to the management of neuromuscular patients who were/are on immunomodulation. This is not only applicable to the COVID-19 pandemic but potentially generalizable to the risk of infections among neuromuscular patients.

An interesting observation for patients not on immunomodulatory agents is that 13% did not exhibit an antibody response, despite not being immunocompromised. Furthermore, only 33% of individuals demonstrated a strong response to the vaccines as defined by antibody level >20.00 µg/mL (see Figures S2–S6, Supplemental Digital Content, http://links.lww.com/MD/O405, which displays subgroup results). The authors found this to be lower than expected and appear to provide a rationale for “booster shots” for the general population.

Overall, among the immunomodulatory agents studied, B-cell depleting agents decrease COVID-19 antibody response the most. Mycophenolate mofetil also attenuates the immunological response but only modestly. Surprisingly, the effect of corticosteroids and IVIG on antibody response was not measurable in this analysis. These observations are similar to findings from other investigators,^[10–13]^ and could be due to corticosteroids and IVIG negligibly affecting the humoral immune system compared to more powerful therapies such as B-cell depleting therapies and mycophenolate mofetil. Moreover, the average age of responders is lower than nonresponders.

There are a number of limitations to this current study. First, there were a small number of patients in our cohort on immunomodulatory agent monotherapy. Second, our use of semi-quantitative antibody responses as a surrogate marker of the human body’s natural immunological response to vaccines may not completely reflect the full immunological response, particularly that of the cellular immune system response. A recent study has shown that rituximab appears to attenuate humoral response to a greater degree than cellular immune response.^[14]^ Another limitation to this analysis is that we assumed that immune responses would not be significantly affected by the patients’ neuromuscular diagnoses. Additionally, there are other co-morbid factors that may affect the semi-quantitative antibody response, and this was a single-site study, so geographic and site-specific practices may introduce uncontrolled biases, including immunomodulatory agents that reflect the medical care at our center. Finally, patients were mostly studied in the early period of the COVID-19 pandemic, and while we made efforts to exclude patients who had COVID-19 infections, we cannot rule out that patient antibody response may have been due to a COVID-19 infection.

## 5. Conclusion

This retrospective chart study may be useful in understanding why some individuals on immunomodulatory treatments may be more susceptible to infections than others. Not surprisingly, when using antibody response to a vaccine, B-cell depleting agents appeared to have the most effect on the immune system and mycophenolate had a modest effect. Somewhat surprisingly, corticosteroids and immunoglobulin treatment had no significant effect. These observations may have implications for clinicians and their patients as they consider different types of immunomodulatory agents. Future research may include prospective studies comparing different immunomodulatory treatments in larger sample sizes and their effect on both cell-mediated immunity as well as the humoral immune system.

## Author contributions

**Data acquisition:** Perry B. Shieh.

**Data curation:** Jaylin Hsu, Perry B. Shieh.

**Formal analysis:** Jaylin Hsu, Perry B. Shieh.

**Supervision:** Perry B. Shieh.

**Writing – original draft:** Jaylin Hsu, Perry B. Shieh.

**Writing – review & editing:** Jaylin Hsu, Perry B. Shieh.

## Supplementary Material


