# Supplementary material for: Effect of immunomodulatory agents on the response to COVID-19 vaccination among patients with neuromuscular diseases: A single center experience
Source: Medicine (Baltimore). 2025 Feb 28;104(9):e41606. doi: 10.1097/MD.0000000000041606 (PMC11875606; doi:10.1097/MD.0000000000041606)
Supplement: Supplementary file 1 [file medi-104-e41606-s001.docx]

**Effect of immunomodulatory agents on the response to COVID-19 vaccination among patients with neuromuscular diseases: A single center experience**

First author: Hsu, Jaylin

**Supplemental Digital Content 1.**

*****On next page for table to fit on full page.

Table 1. Diagnoses, Number of Patients, and Medications Used

| **Diagnosis** | **Number of Patients** | **Immunomodulatory Treatment (s) Used** |
| --- | --- | --- |
| Myasthenia gravis | 41 | Rituximab  Mycophenolate mofetil  Prednisone  Intravenous immunoglobulin |
| Duchenne muscular dystrophy | 5 | Deflazacort |
| Spinal muscular atrophy | 3 | No immunomodulatory treatments |
| Anti-HMGCR myopathy | 3 | Prednisone  Intravenous immunoglobulin |
| Inclusion body myositis | 3 | No immunomodulatory treatments |
| Chronic inflammatory demyelinating polyradiculoneuropathy | 3 | Mycophenolate mofetil  Intravenous immunoglobulin |
| Dermatomyositis | 2 | Rituximab  Mycophenolate mofetil  Prednisone |
| Limb-girdle muscular dystrophy | 1 | No immunomodulatory treatments |
| Hereditary spastic paraplegia | 1 | No immunomodulatory treatments |
| Lambert-Eaton myasthenic syndrome | 1 | No immunomodulatory treatments |
| Autoimmune autonomic ganglionopathy | 1 | Rituximab  Intravenous immunoglobulin |
| Hereditary motor neuropathy | 1 | Intravenous immunoglobulin |
| Congenital muscular dystrophy | 1 | No immunomodulatory treatments |
| Muscular dystrophy | 1 | Intravenous immunoglobulin |

**Effect of immunomodulatory agents on the response to COVID-19 vaccination among patients with neuromuscular diseases: A single center experience**

First author: Hsu, Jaylin

**Supplemental Figure 2.**

**Effect of immunomodulatory agents on the response to COVID-19 vaccination among patients with neuromuscular diseases: A single center experience**

First author: Hsu, Jaylin

**Supplemental Figure 3.**

**Effect of immunomodulatory agents on the response to COVID-19 vaccination among patients with neuromuscular diseases: A single center experience**

First author: Hsu, Jaylin

**Supplemental Figure 4.**

**Effect of immunomodulatory agents on the response to COVID-19 vaccination among patients with neuromuscular diseases: A single center experience**

First author: Hsu, Jaylin

**Supplemental Figure 5.**

**Effect of immunomodulatory agents on the response to COVID-19 vaccination among patients with neuromuscular diseases: A single center experience**

First author: Hsu, Jaylin

**Supplemental Figure 6.**
